# Stiffness modification of two ankle-foot orthosis types to optimize gait in individuals with non-spastic calf muscle weakness – a proof-of-concept study

**DOI:** 10.1186/s13047-019-0348-8

**Published:** 2019-08-07

**Authors:** Hilde E. Ploeger, Niels F. J. Waterval, Frans Nollet, Sicco A. Bus, Merel-Anne Brehm

**Affiliations:** Amsterdam UMC, University of Amsterdam, Rehabilitation, Amsterdam Movement Sciences, Meibergdreef 9, Amsterdam, Netherlands

**Keywords:** Calf muscle weakness, Ankle-foot orthosis, Spring stiffness modification, Gait biomechanics, Walking energy cost, Neuromuscular disorders, Poliomyelitis

## Abstract

**Background:**

To reduce gait problems in individuals with non-spastic calf muscle weakness, spring-like ankle-foot orthoses (AFOs) are often applied, but they are not individually optimized to treatment outcome. The aim of this proof-of-concept study was to evaluate the effects of modifying the stiffness for two spring-like AFO types with shoes-only as reference on gait outcomes in three individuals with calf muscle weakness due to polio.

**Methods:**

We assessed 3D gait biomechanics, walking speed and walking energy cost for shoes-only and five stiffness conditions of a dorsal-leaf-spring AFO and a spring-hinged AFO. Outcomes were compared between stiffness conditions in the two AFOs and three subjects.

**Results:**

Maximum ankle dorsiflexion angle decreased with increasing stiffness in both AFOs (up to 6–8°) and all subjects. Maximum knee extension angle changed little between stiffness conditions, however different responses between the AFOs and subjects were observed compared to shoes-only. Walking speed remained unchanged across conditions. For walking energy cost, we found fairly large differences across stiffness conditions with both AFOs and between subjects (range 3–15%).

**Conclusions:**

Modifying AFO stiffness in individuals with non-spastic calf muscle weakness resulted in substantial differences in ankle biomechanics and walking energy cost with no effect on speed. Our results provide proof-of-concept that individually optimizing AFO stiffness can clinically beneficially improve gait performance.

**Electronic supplementary material:**

The online version of this article (10.1186/s13047-019-0348-8) contains supplementary material, which is available to authorized users.

## Background

In individuals with calf muscle weakness due to neuromuscular disorders, gait is frequently hampered by excessive ankle dorsiflexion and knee flexion during stance, and by reduced ankle push-off [1–6]. This leads to gait problems such as instability, and an increased walking energy cost [2, 6, 7], which may affect functioning in daily life [8].

A spring-like ankle-foot orthosis (AFO) can reduce gait problems in individuals with calf muscle weakness [9–11]. To this purpose, the AFO needs to limit the ankle range of motion in dorsiflexion direction, which restrains excessive ankle dorsiflexion, enables the center of pressure to move forward along the foot and reduces increased knee flexion [2, 3, 11]. Accordingly, this improves ankle and knee stability and reduces walking energy cost, which has been reported in subjects with polio, stroke and MS [2, 6, 12]. Spring-like AFOs also have the ability to store and release energy, thereby supporting ankle push-off [6, 13–16] and taking over ankle work [6, 16], which may even further reduce walking energy cost compared to AFOs without springs.

The effectiveness of spring-like AFOs for calf muscle weakness on gait biomechanics and walking energy cost however is stiffness dependent [17–19] and may differ between individuals [19]. This has been shown in model simulations [17], in healthy subjects [18], and in a small group of subjects with stroke and MS exhibiting calf muscle weakness with spasticity [19]. To our awareness, no studies have been published among conditions with non-spastic calf muscle weakness. The gait pattern from healthy subjects and subjects with stroke and MS without AFO is different from that in polio, and therefore the aims and effects of the AFO are different and, accordingly not evidently transferable.

In clinical practice, two types of spring-like AFOs are currently applied; a dorsal-leaf-spring AFO (DLS-AFO) and a spring-hinged AFO (SH-AFO). The DLS-AFO holds a leaf spring on the dorsal side of the ankle that restrains ankle dorsiflexion but also plantarflexion, particularly at higher stiffness level [19, 20]. The SH-AFO has a hinge on the lateral side that contains a ventral and dorsal unit relative to the ankle joint center in which springs can be inserted, allowing the stiffness towards plantar and dorsiflexion to be adjusted independently. The stiffer the spring (i.e. for the SH-AFO the spring in the ventral unit) the more restriction to ankle dorsiflexion and thus the smaller the maximum ankle dorsiflexion. The highest spring stiffness available for the SH-AFO is much lower than that available for the DLS-AFO, however the SH-AFO springs are functioning as a dorsiflexion stop once they are fully compressed.

Which stiffness level in a particular spring-like AFO is most beneficial for the individual and how large the effect is of varying the stiffness on different gait outcomes is unknown. Knowledge on this matter could facilitate AFO selection and optimize treatment outcome. Therefore, we set up this proof-of-concept study to evaluate the effects of modifying AFO spring stiffness in two different spring-like AFO types on gait in three individuals with non-spastic calf muscle weakness with shoes-only as reference condition. Gait was evaluated in terms of ankle and knee biomechanics and functional measures like comfortable walking speed, walking energy cost, and satisfaction.

## Methods

### Subjects

Three individuals using an AFO for unilateral calf muscle weakness due to poliomyelitis, primarily affecting motor neurons in the spinal cord, who visited our outpatient Rehabilitation clinic, were invited to participate.

**Fig. 1:**
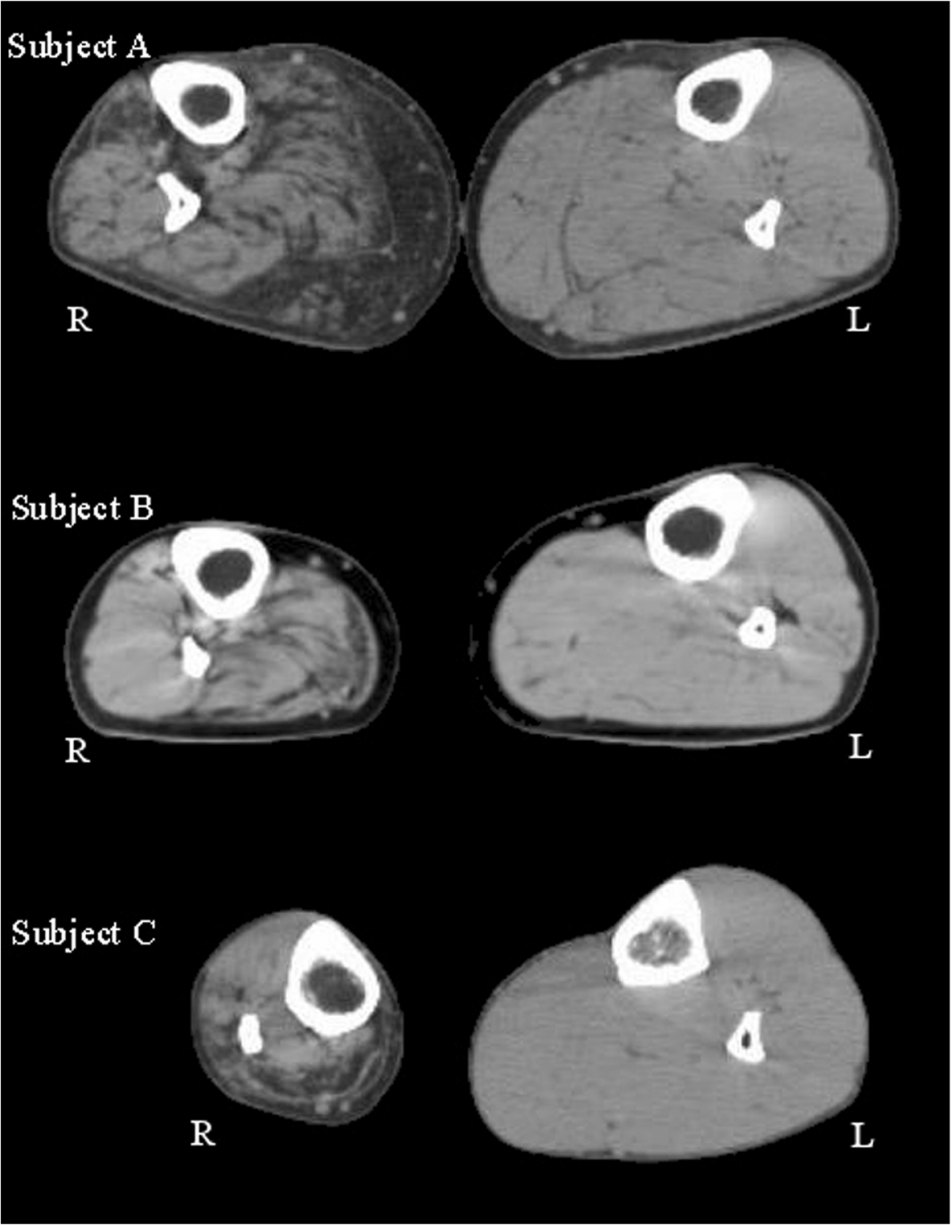
Computed tomography images of the lower right (R) and left (L) leg of the three subjects

We defined calf muscle weakness as a manual muscle strength graded according to the Medical Research Council (MRC) scale < 5 on medical examination [21]. Subject A and B had MRC score 4, and subject C had MRC score 0. When inspecting the computed tomography images of the calf muscles, subject A and B had severely fatty-infiltrated muscles (Fig. 1). Since the MRC scale is insensitive to detect muscle weakness of large muscles groups, calf muscle weakness in subject A and B is more pronounced than indicated by the MRC score [22]. At the knee and hip joints, all subjects had no or mild muscle weakness (MRC 4+ or higher and little fatty-infiltrated muscles) and normal joint mobility (Table 1). Gait of all three subjects when walking without AFO was characteristic of calf muscle weakness [5] and included an increased ankle dorsiflexion and knee flexion during terminal stance and a reduced ankle power during push-off on the affected side compared to normative data of heathy subjects.

**Table 1:** Subject characteristics

	Subject A	Subject B	Subject C
Gender	Male	Male	Male
Age (year)	67	58	60
Length (cm)	173	183	183
Mass (kg)	84	80	65
Side affected	Right	Right	Right
DLS-AFO use (years)	5.9	5.7	1.6
Shoes	Low-cut off the shelf	High-cut orthopedic	Low-cut sports shoes
Heel-sole difference shoe affected side (mm)	15	23	0
Muscle strength, affected side (MRC scores)			
Hip flexors	4+	5	5-
Hip extensors	5	5	5
Hip abductors	5	5	4
Hip adductors	5	5	5
Knee flexors	4+	5	5
Knee extensors	4+	5	5-
Ankle dorsiflexors	3	1	4
Ankle plantarflexors	4	4	0
Passive joint range-of-motion#			
Hip flexion and extension	Normal	Normal	Normal
Knee flexion and extension	Normal	Normal	Normal
Ankle dorsiflexion (degrees)**†**	−10	−10	40
Ankle plantarflexion (degrees)**†**	60	45	−10

#: Joint range-of-motion was measured passively by hand [23]. For ankle range-of-motion measurements the knee was extended

**†:** Minus 10 degrees of dorsiflexion means that dorsiflexion could not be reached manually and the ankle could not be moved further than 10 degrees plantarflexion. Likewise, minus 10 degrees plantarflexion means that the ankle could not move further that 10 degrees dorsiflexion. Note: during weight-bearing, ankle dorsiflexion was possible in subject A and B

Because measurements were done in a patient care setting, the local ethics committee waived the requirement for ethical review of this proof-of-concept study under the Medical Research Involving Human Subjects Act in the Netherlands, although written informed consent was obtained from the subjects prior to the measurements.

### Experimental AFOs

**Fig. 2:**
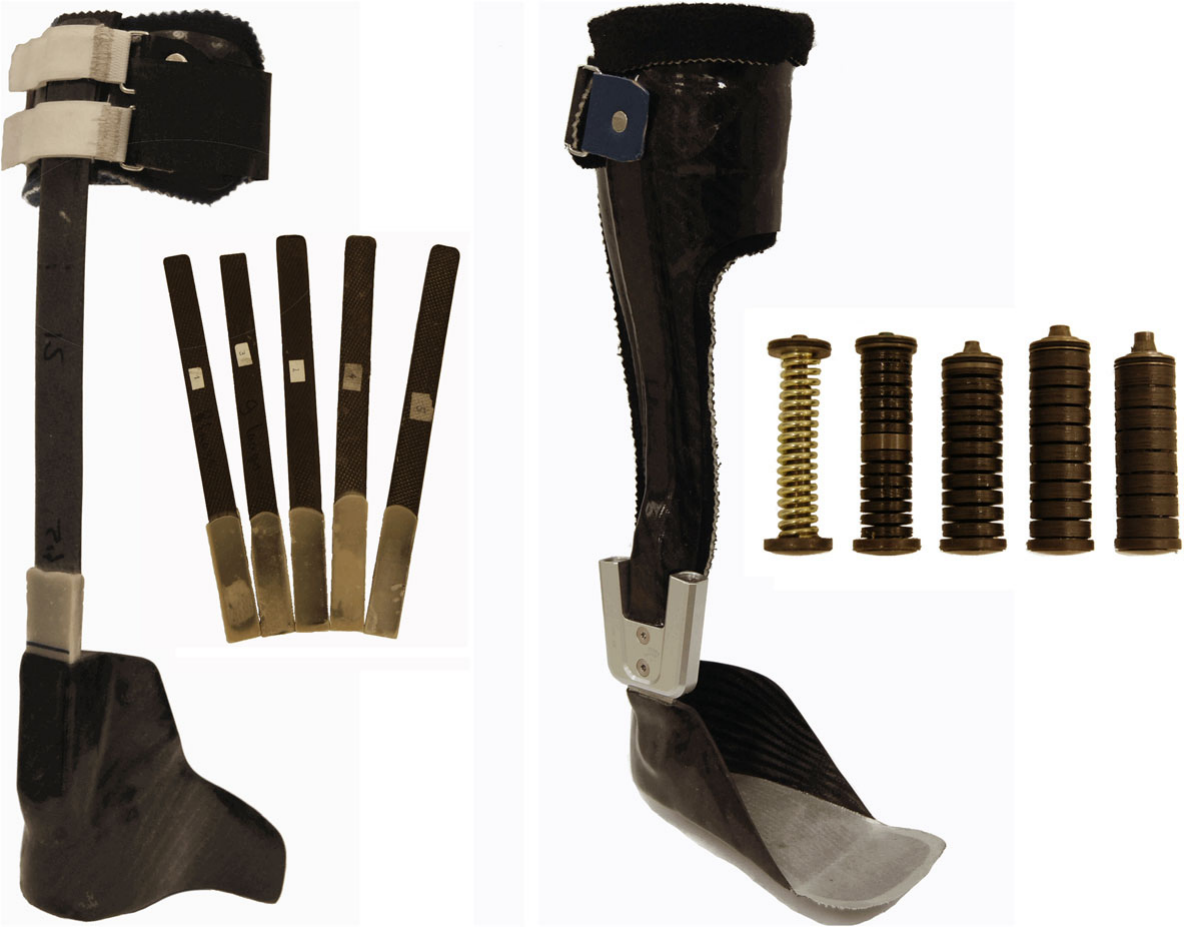
Example of the two types of ankle-foot orthoses Left: the dorsal-leaf-spring ankle-foot-orthosis, and right: the spring-hinged ankle-foot-orthosis, both with their five interchangeable springs with different degrees of stiffness.

For each subject, a foot and calf casing for the DLS-AFO and the SH-AFO (Fig. 2) were custom made from carbon composite by the same orthotist (OIM Orthopedie, Noordwijkerhout, The Netherlands). Furthermore, for the DLS-AFO, the orthotist custom-made five carbon composite springs ranging from most compliant (DLS-k1) to most stiff (DLS-k5), which could be interchanged between the foot and calf casings from the subjects’ DLS-AFO. For the SH-AFO, the 16 mm NeuroSwing® ankle hinge was used (Fior & Gentz, Lüneburg, Germany) that contains a ventral and dorsal spring unit relative to the ankle joint center. Five commercially available springs with different stiffness (from SH-k1: most compliant to SH-k5: most stiff) were tested in the ventral unit (to restrain dorsiflexion). In the dorsal unit, the most compliant spring was used to counteract a possible foot drop, though allowing plantarflexion during stance with little force. No changes in range-of-motion settings of the NeuroSwing® springs were made. Due to foot deformity, a ¾-length instead of a full-length foot plate was used in subject B. For all three subjects, the same springs were used throughout all tests. The AFOs were built in neutral with the tibia 2 degrees inclined anterior, taking into account the heel-sole difference from the subjects’ shoes. There was no heel-sole difference of the AFOs foot plates. The AFOs were worn in the same shoe as during the shoes-only condition in order to keep the heel-sole difference similar between all conditions since changing the heel-sole difference can affect gait biomechanics [24].

### Procedures

DLS-AFO and SH-AFO conditions were tested on two separate days. Gait biomechanics and patient satisfaction were assessed for the five stiffness conditions per AFO and for shoes-only. To limit the burden on subjects, comfortable walking speed and walking energy cost were only assessed for three stiffness conditions per AFO and for shoes-only. For most contrast, we chose the most compliant (k1), the stiffest (k5) and the spring with stiffness value closest to the middle, which was k3 for the DLS-AFO and k4 for the SH-AFO (Table 2).

**Table 2:** Spring stiffness k in N•m•deg^− 1^ (mean (SD))

	DLS-AFO	SH-AFO
k1 (most compliant)	0.9 (0.1)	0.05 (0.00)
k2 (moderately compliant)	1.4 (0.2)	0.3 (0.04)
k3 (medium stiff)	2.7 (0.2)	0.8 (0.1)
k4 (moderately stiff)	5.7 (0.2)	1.4 (0.3)
k5 (stiffest)	7.2 (0.1)	2.2 (0.4)

Abbreviations: DLS-AFO: dorsal-leaf-spring ankle-foot-orthosis, SH-AFO: spring-hinged ankle-foot-orthosis

### Measurements

#### Stiffness properties of the experimental AFOs

Stiffness properties of both AFOs were measured with the Bi-articular Reciprocal Universal Compliance Estimator (BRUCE) for all five springs [25]. See Bregman et al. [25] and Kerkum et al. [26] for procedures. Briefly, the calf casing of the AFO (DLS and SH) was fixated to BRUCE’s dummy leg with a velcro strap in such a way the rotational axis was aligned with the ankle axis of BRUCE. Per spring condition, the DLS-AFO was manually fully compressed towards dorsiflexion and plantarflexion, and the SH-AFO was fully compressed towards dorsiflexion and slowly released back to neutral. The BRUCE device simultaneously measured the ankle angle and exerted moment on the device. Each spring condition was tested three times.

#### Gait biomechanics

Gait biomechanics were assessed while walking at self-selected comfortable speed along a 12-m long walkway using a 3D eight-camera system operating at 100 Hz (VICON MX1.3) and a conventional gait model (Plug-in Gait, VICON, Oxford, UK). Simultaneously, the ground reaction force was recorded using two force plates in series lying flush with the floor operating at 1000 Hz (OR6–7, AMTI, Watertown, MA, USA).

Measurements for each condition were repeated until five steps of both legs were recorded within the force plate borders and markers visible by the optical cameras from heel strike on the force plate to subsequent ipsilateral heel strike. The shoes-only condition was measured on both testing days and markers were left on the body until all conditions were finished, to minimize differences in marker placement. Conditions per testing day were performed in random order. For logistic reasons, one subject had both AFOs tested on one day. He could endure the tests well and had no physical complaints. Subjects were given as much time as needed between measurements to rest and customize to each new condition if needed.

#### Walking speed and walking energy cost

Walking speed and walking energy cost were measured during a 6-min walking energy cost test at self-selected comfortable speed [27] on an oval 35-m indoor track. Throughout the test, oxygen-uptake and respiratory exchange ratios were assessed with a telemetric gas analysis device (Cosmed K4b2, Rome, Italy) [28]. Subjects had 15 min of rest (sitting on a chair) between the conditions.

#### Satisfaction

Satisfaction about walking was rated directly after each gait analysis condition using a numeric rating scale (NRS), with scores ranging from 0 (least possible satisfaction) to 10 (highest possible satisfaction). In addition, subjects were asked to clarify their answer.

### Data analysis

#### Stiffness properties

Per AFO type, for each stiffness condition, the ankle angle and exerted ankle moment as measured with BRUCE were plotted using a custom written Matlab script (version 2011, The MathWorks, Inc., Natick, MA, USA). Over the spring’s linear compression phase, a linear fit was plotted and the slope of this line was used to calculate the stiffness (N•m•deg^− 1^) determined as: change in ankle moment divided by change in ankle angle [25, 26]. Subsequently, the mean and standard deviation were calculated from nine (3 repetitions x 3 AFOs (from each individual)) stiffness values of each AFO spring condition.

#### Gait biomechanics

Per condition, five valid trials were processed with standard Plug-In-Gait pipelines (VICON Nexus 1.8.5, Oxford, UK) to determine spatiotemporal parameters, and joint angles (degrees), moments (N•m•kg^− 1^) and powers (Watt•kg^− 1^) of the ankle and knee. Heel-sole difference was set at 0 for all trials for comparative purposes, meaning that the ankle angle represented the angle between the lower leg and the shoe sole. The ankle angle in the shoe was for subject A 4° plantarflexion, for subject B 7° plantarflexion and for subject C neutral. Trials were time-normalized with spline interpolation and means and standard deviations were calculated using a custom written Matlab script (version R2014a, The MathWorks, Inc., Natick, MA, USA).

Kinematic and kinetic parameters of the ankle and knee were examined, with focus on minimum sagittal plane ankle angle during loading response, and maximum sagittal plane ankle angle, maximum ankle power and minimum sagittal plane knee angle during stance [2, 6]. Minimum and maximum ankle angles were calculated relative to the angle at initial contact to account for differences in AFO alignment and marker placement between subjects.

#### Walking speed and waking energy cost

Comfortable walking speed was calculated as the average speed over 6 min of walking. Mean steady-state breath-by-breath oxygen uptake values and respiratory exchange ratios were computed over the last three minutes of the walking energy cost test and used to calculate gross walking energy consumption (J•kg^− 1^•min^− 1^) [29]. Gross energy consumption was divided by steady state speed (m•s^− 1^) to calculate the gross walking energy cost (J•kg^− 1^•m^− 1^) [7].

### Statistics

Due to the small sample size, we only reported descriptive data on gait biomechanics, walking speed, walking energy cost and satisfaction, which we compared between shoes-only and all stiffness conditions of both AFO types and the three subjects**.**

## Results

### Experimental AFO stiffness properties

Stiffness of the DLS-AFO ranged from 0.9 (SD 0.1) to 7.2 (SD 0.1) N•m•deg^− 1^, and stiffness of the SH-AFO from 0.05 (SD 0.0) to 2.2 (SD 0.4) N•m•deg^− 1^ (Table 2).

### Gait biomechanics

#### Sagittal plane ankle angle

**Fig. 3:**
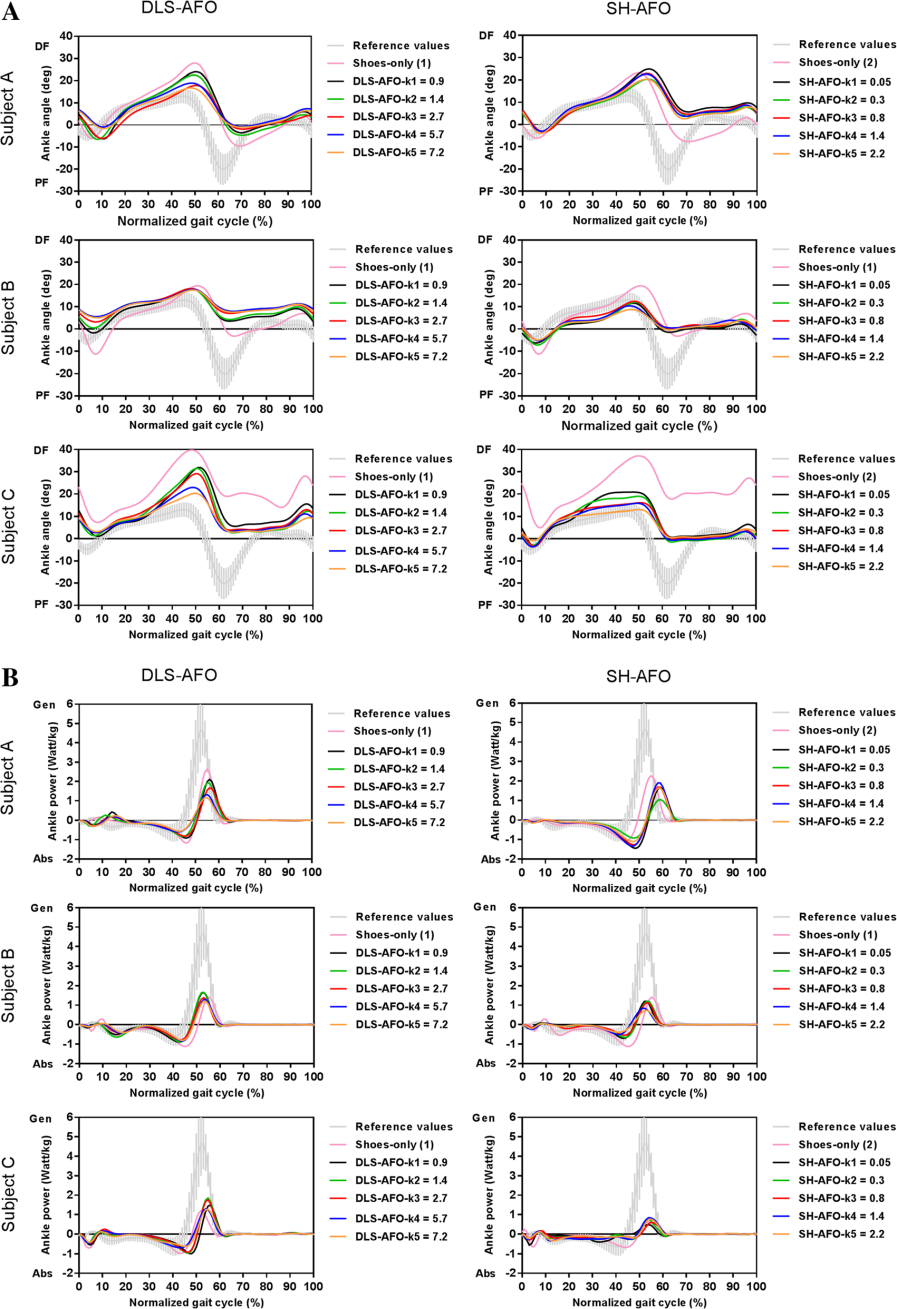

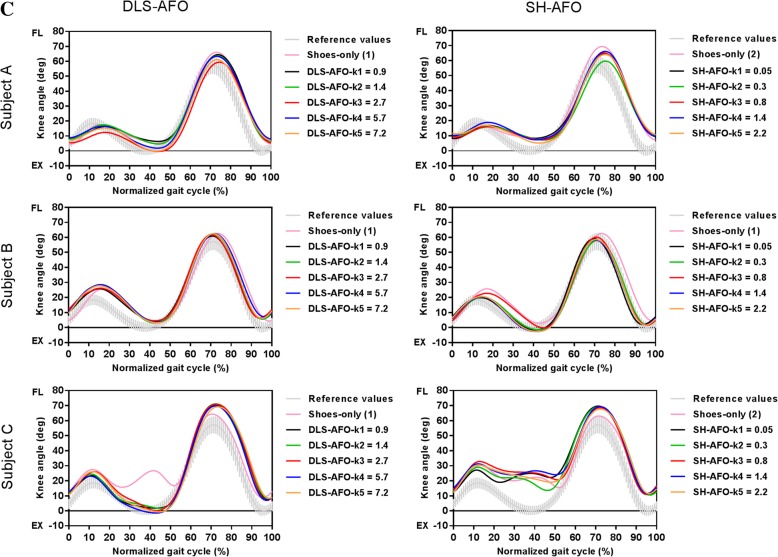
Gait biomechanics. (**a**) Ankle angles, (**b**) ankle powers, (**c**) knee angles of subject A (calf muscle strength MRC 4), subject B (calf muscle strength MRC 4) and subject C (calf muscle strength MRC 0). Shoes-only (1) is performed at the DLS-AFO testing day, Shoes-only (2) is performed at the SH-AFO testing day (subject B had all AFO conditions tested at one day, therefore only one shoes-only condition was performed). Abbreviations: DLS-AFO: dorsal-leaf-spring ankle-foot-orthosis, SH-AFO: spring-hinged ankle-foot-orthosis, k: stiffness in N•m•deg^− 1^, DF: dorsiflexion, PF: plantarflexion, EX: extension, FL: flexion, Gen: generation, Abs: absorption

Maximum ankle angle (dorsiflexion) during terminal stance was smaller in almost all stiffness conditions of both AFOs, and for all three subjects when compared to shoes-only, and decreased with increasing stiffness (k1-k5) between 5 and 8° for the DLS-AFO and between 2 and 6° for the SH-AFO (Fig. 3a, Table 3).

**Table 3:** Data on 3D gait biomechanics and satisfaction

	Shoes-only1	DLS-k1	DLS-k2	DLS-k3	DLS-k4	DLS-k5	Shoes-only2	SH-k1	SH-k2	SH-k3	SH-k4	SH-k5
Speed (m•s^−1^)												
Subject A	1.11 (0.03)	1.12 (0.04)	1.14 (0.03)	1.12 (0.04)	1.15 (0.03)	1.11 (0.02)	1.07 (0.02)	1.02 (0.03)	0.93 (0.04)	1.01 (0.04)	1.02 (0.04)	1.01 (0.02)
Subject B	1.27 (0.02)	1.32 (0.02)	1.35 (0.02)	1.28 (0.03)	1.31 (0.01)	1.33 (0.02)	N/A	1.20 (0.02)	1.24 (0.05)	1.13 (0.02)	1.25 (0.02)	1.20 (0.01)
Subject C	1.45 (0.03)	1.50 (0.04)	1.49 (0.05)	1.50 (0.03)	1.55 (0.05)	1.55 (0.04)	1.43 (0.03)	1.34 (0.05)	1.38 (0.04)	1.33 (0.03)	1.31 (0.03)	1.27 (0.04)
Maximum ankle angle stance (°)†; positive values are dorsiflexion, negative values plantarflexion												
Subject A	25.9 (1.1)	18.8 (1.1)	18.8 (0.6)	13.8 (0.7)	12.1 (0.7)	10.4 (0.8)	23.6 (0.7)	15.7 (0.5)	13.9 (1.3)	14.7 (1.6)	15.5 (0.9)	13.5 (1.3)
Subject B	15.7 (1.0)	14.1 (0.7)	12.6 (0.7)	10.9 (0.5)	8.9 (0.3)	9.0 (0.3)	N/A	13.5 (0.8)	12.2 (1.2)	12.1 (1.1)	9.4 (1.6)	8.0 (0.4)
Subject C	16.6 (2.3)	17.7 (1.2)	22.1 (1.5)	17.3 (0.9)	14.4 (1.9)	10.9 (1.5)	12.3 (3.7)	16.1 (2.8)	17.0 (0.7)	13.6 (0.6)	15.1 (2.2)	10.1 (1.0)
Minimum ankle angle loading response (°)†; positive values are dorsiflexion, negative values plantarflexion												
Subject A	−8.0 (1.0)	−11.9 (1.9)	−10.4 (1.5)	−10.0 (1.3)	−8.0 (0.8)	−8.1 (0.9)	−5.9 (1.2)	−9.7 (0.5)	−7.8 (0.9)	−9.8 (0.9)	−8.7 (0.5)	−9.7 (2.0)
Subject B	−14.8 (1.1)	−5.9 (1.0)	−5.1 (0.9)	−4.1 (0.6)	−3.4 (0.2)	−3.5 (0.6)	N/A	−4.6 (0.9)	−7.3 (1.7)	−5.4 (0.1)	−6.4 (1.7)	−5.9 (0.5)
Subject C	−15.9 (4.9)	−11.8 (1.9)	−7.5 (0.8)	−8.9 (1.4)	−6.4 (2.1)	−6.2 (0.8)	−20.1 (4.0)	−8.6 (3.8)	−4.9 (1.0)	−5.8 (1.5)	−4.6 (1.3)	−3.8 (1.4)
Maximum internal ankle moment single stance (N•m•kg^− 1^); positive values are plantarflexion, negative values dorsiflexion												
Subject A	1.00 (0.04)	1.05 (0.06)	1.07 (0.05)	1.11 (0.06)	1.12 (0.02)	1.08 (0.03)	1.10 (0.04)	1.14 (0.10)	0.95 (0.07)	1.16 (0.13)	1.32 (0.10)	1.33 (0.06)
Subject B	1.07 (0.04)	1.25 (0.02)	1.26 (0.03)	1.26 (0.02)	1.27 (0.02)	1.26 (0.03)	N/A	0.81 (0.04)	0.85 (0.02)	0.80 (0.002)	0.82 (0.02)	0.80 (0.02)
Subject C	0.91 (0.06)	0.64 (0.03)	0.76 (0.08)	0.79 (0.10)	0.90 (0.06)	0.98 (0.06)	0.75 (0.11)	1.29 (0.05)	1.19 (0.12)	1.11 (0.09)	1.10 (0.07)	1.11 (0.06)
Maximum ankle power single stance (Watt•kg^−1^); positive values are power generation, negative values absorption												
Subject A	2.6 (0.2)	2.2 (0.2)	2.1 (0.2)	1.8 (0.3)	1.4 (0.2)	1.3 (0.1)	2.3 (0.2)	1.7 (0.2)	1.1 (0.1)	1.7 (0.2)	1.9 (0.2)	1.7 (0.2)
Subject B	1.5 (0.1)	1.7 (0.1)	1.7 (0.1)	1.4 (0.1)	1.3 (0.1)	1.2 (0.1)	N/A	1.2 (0.2)	1.2 (0.1)	1.1 (0.1)	0.9 (0.1)	0.8 (0.1)
Subject C	1.2 (0.2)	1.5 (0.1)	1.9 (0.2)	1.9 (0.3)	1.5 (0.2)	1.4 (0.1)	0.7 (0.3)	0.6 (0.04)	0.8 (0.2)	0.6 (0.04)	0.9 (0.1)	0.8 (0.1)
Minimum knee angle single stance (°); positive values are flexion, negative values extension												
Subject A	5.1 (0.9)	6.1 (1.4)	4.3 (1.1)	−0.4 (0.4)	1.7 (0.4)	−0.2 (0.5)	7.2 (0.8)	8.1 (0.7)	7.1 (0.9)	7.3 (1.2)	7.3 (1.5)	5.1 (0.6)
Subject B	2.8 (0.5)	4.0 (0.2)	3.1 (0.6)	4.4 (0.6)	3.3 (0.2)	3.2 (0.6)	N/A	−1.7 (0.5)	−1.5 (0.3)	−0.6 (2.7)	−2.7 (0.5)	−2.7 (0.3)
Subject C	16.8 (1.3)	1.6 (0.8)	1.4 (2.0)	−0.1 (0.7)	−1.6 (0.5)	−1.0 (4.0)	16.4 (3.7)	19.5 (7.2)	12.6 (10.3)	20.5 (3.8)	19.8 (7.4)	18.8 (6.8)
Maximum knee angle early stance (°); positive values are flexion, negative values extension												
Subject A	17.6 (2.7)	16.5 (1.0)	17.6 (2.8)	12.4 (1.2)	16.5 (2.4)	15.4 (1.1)	16.3 (1.7)	16.9 (1.7)	16.1 (1.7)	16.2 (0.7)	19.0 (2.0)	17.1 (0.3)
Subject B	25.8 (1.3)	25.9 (1.5)	28.5 (1.0)	26.3 (1.8)	28.6 (1.1)	27.8 (1.3)	N/A	19.9 (0.6)	20.4 (1.6)	22.8 (2.8)	20.6 (1.5)	20.8 (1.0)
Subject C	27.6 (1.5)	23.7 (3.5)	24.9 (4.6)	26.3 (3.1)	23.2 (1.7)	26.1 (2.9)	30.3 (4.2)	27.5 (3.2)	29.1 (2.2)	33.0 (4.0)	31.4 (3.5)	30.6 (5.5)
Minimum internal knee moment single stance (N•m•kg^−1^); positive values are extension, negative flexion												
Subject A	0.14 (0.03)	0.14 (0.07)	0.10 (0.08)	−0.09 (0.05)	−0.03 (0.04)	−0.07 (0.07)	0.10 (0.03)	0.07 (0.03)	0.06 (0.07)	0.04 (0.04)	0.02 (0.05)	−0.07 (0.03)
Subject B	−0.17 (0.05)	−0.20 (0.03)	−0.27 (0.03)	−0.17 (0.04)	−0.25 (0.01)	−0.22 (0.05)	N/A	−0.15 (0.03)	−0.17 (0.01)	−0.10 (0.19)	−0.22 (0.04)	−0.21 (0.03)
Subject C	0.33 (0.10)	0.09 (0.03)	0.06 (0.07)	−0.01 (0.04)	−0.12 (0.05)	−0.19 (0.14)	0.34 (0.04)	0.25 (0.09)	0.09 (0.20)	0.25 (0.07)	0.27 (0.15)	0.22 (0.16)
Satisfaction about walking (NRS score)§												
Subject A	4	5	5	7*	2	6	3	5	5	5	4	5
Subject B	4	6	6	6.5	7	7	N/A	5	8*	6	6	7
Subject C	5	4	3	7	6	8*	6	2	3	4	4	6

Subject A: calf muscle strength MRC 4, subject B: calf muscle strength MRC 4, subject C: calf muscle strength MRC 0. Gait biomechanics data are expressed as mean (SD). Shoes-only1 is performed at the DLS-AFO testing day, Shoes-only2 is performed at the SH-AFO testing day (subject B had all AFO conditions tested at one day, therefore only one shoes-only condition was performed)

Loading response: 1–10% gait cycle, early stance: 1–20% gait cycle, †: plantar and dorsiflexion angles are relative to the ankle angle at initial contact, §: satisfaction about walking in general when walking on shoes-only and with different AFO stiffness scored on a numeric rating scale (NRS) with 10 as highest satisfaction, *: quotes about the most satisfying condition: subject A: “was just sufficient for my remaining muscle force; it supports, but obstructs not too much”. Subject B: “it gives support, but not annoyingly. I like the design of the SH-AFO, the ankle feels not completely blocked in an angle of 90 degrees”. Subject C about all SH-AFO conditions and DLS-AFO-k1 and k2: “too compliant, does hardly anything”. About DLS-AFO-k3 he said: “support starts to increase”, and about the DLS-AFO-k5 “giving the best support”

Abbreviations: DLS: dorsal-leaf-spring ankle-foot-orthosis, SH: spring-hinged ankle-foot-orthosis, k: stiffness (from most compliant (k1) to most stiff (k5)), N/A: not applicable

Minimum ankle angle (plantarflexion) during loading response was smaller in all AFO conditions compared to shoes-only in subject A and larger in subject B and C (Fig. 3a, Table 3). Minimum ankle angle during loading response increased with increasing stiffness (k1 to k5) between 2 and 6° for the DLS-AFO, while for the SH-AFO only in subject C, an increase from k1 to k5 (3°) was found.

#### Ankle power

With the DLS-AFO, maximum ankle power was lower compared to shoes-only in all stiffness conditions in subject A, and in the three stiffest conditions in subject B (Fig. 3b, Table 3). In subject B (two most compliant conditions) and C (all stiffness conditions) maximum ankle power was higher compared to shoes-only. In all three subjects, maximum ankle power decreased between 0.5–0.9 W•kg^− 1^ with increasing DLS-AFO stiffness (k1 to k5).

With the SH-AFO, maximum ankle power was lower compared to shoes-only in all stiffness conditions in subject A and B, and was not different in subject C (Fig. 3b, Table 3). Maximum ankle power decreased with increasing SH-AFO stiffness (k1 to k5) only in subject B (with 0.4 W•kg^− 1^).

#### Sagittal plane knee angle

With the DLS-AFO, minimum knee angle during stance was between 15° and 18° smaller compared to shoes-only for all stiffness conditions in subject C, and for the three stiffest conditions, between 3° and 5°, in subject A (both subjects reaching full extension) (Fig. 3c, Table 3). In subject B, the minimum knee angle remained in 3 or 4 degrees flexion when walking with all stiffness conditions.

With the SH-AFO, no differences in minimum knee angle during single stance compared to shoes-only were found in subject A and C, while in subject B the SH-AFO decreased the minimum knee angle in all stiffness conditions, between 3° to 6°, compared to shoes-only (reaching full extension) (Fig. 3c, Table 3).

See additional figures for additional gait biomechanical parameters (Additional files 1, 2 and 3).

### Walking speed and walking energy vcost

In subject A, comfortable walking speed was higher compared to shoes-only in all three DLS-AFO conditions (between + 5 and + 11%) (Table 4), and remained unchanged in all SH-AFO condition. In subject B and C, walking speed changed little between stiffness conditions in both AFOs (from − 4 to + 2%).

**Table 4:** Self-selected comfortable walking speed and walking energy cost and during the 6-min walk test

	Shoes-only1	DLS-k1	DLS-k3	DLS-k5	Shoes-only2	SH-k1	SH-k4	SH-k5
Speed (m•s^− 1^)								
Subject A	1.08	1.14 (+6%)	1.19 (+11%)	1.13 (+5%)	1.12	1.11 (−1%)	1.12 (+0.4%)	1.11 (−0.4%)
Subject B	1.26	1.25 (−1%)	1.21 (−3%)	1.28 (+2%)	N/A	1.25 (−1%)	1.21 (−4%)	1.23 (−2%)
Subject C	1.44	1.45 (+0.3%)	1.40 (−3%)	1.48 (+2%)	1.49	1.43 (−4%)	1.46 (−2%)	1.47 (−2%)
Walking energy cost (J•kg^−1^•min^−1^)								
Subject A	4.22	4.16 (−2%)	4.07 (−4%)	4.03 (−5%)	5.02	4.98 (−1%)	4.49 (−11%)	5.03 (+0.1%)
Subject B	4.71	4.33 (−8%)	4.20 (−11%)	4.82 (+2%)	N/A	4.17 (−11%)	3.86 (−18%)	4.44 (−6%)
Subject C	4.73	5.00 (+6%)	4.54 (−4%)	4.36 (−8%)	4.73	4.47 (−6%)	4.48 (−5%)	4.69 (−1%)

Subject A: calf muscle strength MRC 4, subject B: calf muscle strength MRC 4, subject C: calf muscle strength MRC 0

Shoes-only1 is the shoes-only condition at the DLS-AFO testing day, Shoes-only2 is the shoes-only condition at the SH-AFO testing day (subject B had all AFO conditions tested at one day, therefore only one shoes-only condition was performed)

Percentages are relative compared to shoes-only

Abbreviations: DLS: dorsal-leaf-spring ankle-foot-orthosis, SH: spring-hinged ankle-foot-orthosis, k: stiffness (from most compliant (k1) to most stiff (k5)), N/A: not applicable

For most but not all stiffness conditions of both AFOs, walking energy cost was lower compared to shoes-only (range DLS-AFO: + 6% to − 11%, range SH-AFO: 0% to − 18%) (Table 4). No clear pattern was observed across the subjects with modifying the stiffness. When comparing walking energy cost between the least and most energy effective stiffness condition, a difference of 3–15% with the DLS-AFO and of 5–15% with the SH-AFO was found (Table 4).

### Satisfaction

Satisfaction scores compared to shoes-only showed no clear pattern with modifying the stiffness (between − 2 and + 3 NRS points (DLS-AFO) and − 3 and + 4 NRS points (SH-AFO), Table 4). The difference between the most and least satisfying stiffness was 1–5 NRS points (DLS-AFO) and 1–4 NRS points (SH-AFO). Subjects could clearly clarify their scores (Table 4).

## Discussion

This study in three individuals with calf muscle weakness due to polio showed that modifying the stiffness of spring-like AFOs substantially affected ankle angle, up to 8 degrees, walking energy cost, up to 15%, and satisfaction, up to 5 NRS points. In all subjects, a stiffer AFO reduced the ankle angle more compared to a compliant one, while for the other gait outcomes the effect of stiffness modification differed between subjects and AFOs. Our proof-of-concept study demonstrates that modification of AFO stiffness largely affected gait in individuals with non-spastic calf muscle weakness, with clear beneficial effects on ankle biomechanics, walking energy cost and satisfaction.

Maximum ankle angle (dorsiflexion) is the only gait parameter that responded similarly to modifying AFO stiffness in both AFOs. The reduction of maximal ankle angle with increasing stiffness supports the biomechanical aim (i.e. to restrict dorsiflexion, and to restrict more with higher stiffness), which is in line with results from studies in children with CP [30] using a SH-AFO, and in adults with neurological disorders [19] and trauma [20] using a DLS-AFO. Furthermore, the differences up to 8 degrees in maximal ankle angle between stiffness conditions show that AFO stiffness is highly relevant in this regard and can be used to individually tune the ankle angle. With the stiffest SH-AFO, ankle dorsiflexion reduced to a similar extent as with the stiffest DLS-AFO, despite the lower stiffness. This difference can be attributed to the dorsiflexion stop inherent to the SH-AFO’s spring properties, which was clearly visible in the ankle angle of subject C where ankle dorsiflexion plateaued from mid-stance onwards.

Regarding the other ankle parameters, modifying stiffness showed substantial larger effects in the DLS-AFO compared to the SH-AFO. Increasing stiffness with the DLS-AFO seems to reduce the ankle plantarflexion angle and decrease the ankle power, which is in line with previous studies in other patient groups [18, 19, 31], while for the SH-AFO effects are less clear. The SH-AFO was expected to result in more plantarflexion during loading response compared to the DLS-AFO. However, maximum plantarflexion with the SH-AFO was similar to the DLS-AFO. Even the stiffest DLS-AFO allowed between 4 to 8 degrees plantarflexion, and was apparently less restrictive than expected based on its design, possibly due to an adequate external plantarflexion moment.

That ankle power reduced with increasing stiffness for the DLS-AFO can be explained by the increasing restriction of the ankle range of motion, which reduces the ability to generate power [18, 19, 31]. As ankle power is essential for an energy efficient gait pattern, this should be taken into account when providing an DLS-AFO. That no effect of stiffness was seen with the SH-AFO and maximum values were considerably lower compared to the DLS-AFO can be explained by the relatively low stiffness of the SH-AFO springs and the small dorsiflexion range-of-motion over which the springs could be compressed. This may have resulted in less energy storage and return compared to the DLS-AFOs, thus contributing less to maximum ankle power [17]. Together with the small stiffness range of the springs in the SH-AFO, this may also explain why ankle power did not decrease with increasing stiffness.

At the knee, the minimum knee angle (extension) during stance changed little across stiffness conditions, which agrees with earlier reports [19, 30, 31]. Possibly, the knee extends once a sufficient stiffness is reached. Likely, increasing the stiffness of the AFO will not affect the knee angle further, as more extension is anatomically not possible. Knee extension effects of both AFOs resulted from a reduced ankle dorsiflexion angle, which induced the knee to extend, as well as from improved forward center of pressure excursion that moved the ground reaction force in front of the knee, thereby creating an external knee extension moment [11, 32]. Interestingly, we found large differences in knee angles between AFO types; i.e. either the DLS-AFO increased knee extension while the SH-AFO did not, and almost independently of stiffness, or vice versa. Possibly, other factors than stiffness contributed to the inability to improve knee extension, such as a suboptimal alignment [1, 13] or an inadequate footplate stiffness [33].

Comfortable walking speed changed only little between conditions, which is in line with other studies varying AFO stiffness [19, 30, 34]. Regarding walking energy cost, we found substantial differences up to 15% across stiffness conditions with both AFOs, which were larger than the reported smallest detectable change of 9.4% [7] and considerably larger than the effect of providing standard AFOs in individuals with prior polio [2, 7]. This indicates the clinical relevance of stiffness modification, and confirms that walking energy cost can noticeably be reduced by adjusting AFO stiffness [17–19]. That the stiffness with the lowest walking energy cost differed between subjects, is likely caused by differences in subject characteristics (e.g. calf muscle strength, mass, walking speed) [19]. Future larger studies should investigate the influence of subject characteristics on walking energy cost and the relation between walking energy cost and gait biomechanics, thereby comparing spring-like AFOs to AFOs without a spring to discriminate between the energy storing and biomechanical effects of spring-like AFOs on walking energy cost.

In all three subjects, the most satisfying AFO had good overall gait performance, both in terms of gait biomechanics and walking energy cost. However, AFO stiffness conditions that did not differ in effect were rated considerably different in terms of satisfaction (for example when comparing the three stiffest DLS-AFOs in subject A), indicating that for AFO optimization, qualitative measures are also important to consider.

The short accustomization time to the AFOs can be considered a limitation of this study. For gait biomechanics, accustomization may not be necessary [35]. However, walking energy cost may reduce after accustomization, especially with stiffer AFOs, as co-contraction is more likely to occur when the subject is not yet accustomed and muscle lengths have not yet been adapted [18, 36]. For satisfaction, sufficient accustomization may also be necessary. That we did not control for differences in walking speed between gait biomechanics conditions can be considered a second limitation, as it may have influenced the effects on gait biomechanics and walking energy cost.

## Conclusions

Our proof-of-concept study demonstrates that modification of AFO stiffness largely affected gait in individuals with non-spastic calf muscle weakness, with clear beneficial effects on ankle biomechanics, walking energy cost and satisfaction. However, the stiffness with the largest beneficial effects differed between subjects and AFO types, indicating the relevance of individually customizing AFOs. This should be confirmed in a larger study, also investigating the influence of patient characteristics on optimal AFO stiffness and AFO type to improve gait performance.

## Additional files

Additional file 1: Gait biomechanics of subject A (calf muscle strength MRC 4). Shoes-only (1) is performed at the DLS-AFO testing day, Shoes-only (2) is performed at the SH-AFO testing day. Abbreviations: DLS-AFO: dorsal-leaf-spring ankle-foot-orthosis, SH-AFO: spring-hinged ankle-foot-orthosis, k: stiffness in N•m•deg^− 1^, DF: dorsiflexion, PF: plantarflexion, EX: extension, FL: flexion, Gen: generation, Abs: absorption, CoP: center of pressure. (TIF 1005 kb)

Additional file 2: Gait biomechanics of subject B (calf muscle strength MRC 4). Since all AFO conditions were tested at one day there is only one shoes-only (Shoes-only (1)) condition performed. Abbreviations: DLS-AFO: dorsal-leaf-spring ankle-foot-orthosis, SH-AFO: spring-hinged ankle-foot-orthosis, k: stiffness in N•m•deg^− 1^, DF: dorsiflexion, PF: plantarflexion, EX: extension, FL: flexion, Gen: generation, Abs: absorption, CoP: center of pressure. (TIF 995 kb)

Additional file 3: Gait biomechanics of subject C (calf muscle strength MRC 0). Shoes-only (1) is performed at the DLS-AFO testing day, Shoes-only (2) is performed at the SH-AFO testing day. Abbreviations: DLS-AFO: dorsal-leaf-spring ankle-foot-orthosis, SH-AFO: spring-hinged ankle-foot-orthosis, k: stiffness in N•m•deg^− 1^, DF: dorsiflexion, PF: plantarflexion, EX: extension, FL: flexion, Gen: generation, Abs: absorption, CoP: center of pressure. (TIF 1057 kb)

## Data Availability

All data generated or analyzed during this study are included in this published article and its supplementary information files.

## Abbreviations

AFO: Ankle-foot orthosis
BRUCE: Bi-articular Reciprocal Universal Compliance Estimator
DLS-AFO: Dorsal-leaf-spring ankle-foot orthosis
k: Stiffness
MRC: Medical Research Council
NRS: Numeric Rating Scale
SH-AFO: Spring-hinged ankle-foot orthosis

## References

[1] Perry J, Burnfield JM (2010). Gait analysis, Normal and pathological function.

[2] Ploeger HE, Bus SA, Brehm MA, Nollet F (2014). Ankle-foot orthoses that restrict dorsiflexion improve walking in polio survivors with calf muscle weakness. Gait Posture.

[3] Beekman C, Perry J, Boyd LA, Newsam CJ, Mulroy SJ (2000). The effects of a dorsiflexion-stopped ankle-foot orthosis on walking in individuals with incomplete spinal cord injury. Top Spinal Cord Inj Rehabil.

[4] Sutherland DH, Cooper L, Daniel D (1980). The role of the ankle plantar flexors in normal walking. J Bone Joint Surg Am.

[5] Ploeger HE, Bus SA, Nollet F, Brehm MA (2017). Gait patterns in association with underlying impairments in polio survivors with calf muscle weakness. Gait Posture.

[6] Bregman DJ, Harlaar J, Meskers CG, de Groot V (2011). Spring-like ankle foot orthoses reduce the energy cost of walking by taking over ankle work. Gait Posture.

[7] Brehm MA, Nollet F, Harlaar J (2006). Energy demands of walking in persons with postpoliomyelitis syndrome: relationship with muscle strength and reproducibility. Arch Phys Med Rehabil.

[8] Jensen MP, Alschuler KN, Smith AE, Verrall AM, Goetz MC, Molton IR (2011). Pain and fatigue in persons with postpolio syndrome: independent effects on functioning. Arch Phys Med Rehabil.

[9] Perry J, Clark D (1997). Biomechanical abnormalities of post-polio patients and the implications for orthotic management. NeuroRehabilitation.

[10] Nollet F, Noppe CT, Hsu JD, Michael JW, Fisk JR (2008). Orthoses for persons with postpolio syndrome. AAOS atlas of orthoses and assistive devices.

[11] Lehmann JF, Condon SM, de Lateur BJ, Smith JC (1985). Ankle-foot orthoses: effect on gait abnormalities in tibial nerve paralysis. Arch Phys Med Rehabil.

[12] Danielsson A, Sunnerhagen KS (2004). Energy expenditure in stroke subjects walking with a carbon composite ankle foot orthosis. J Rehabil Med.

[13] Wolf SI, Alimusaj M, Rettig O, Doderlein L (2008). Dynamic assist by carbon fiber spring AFOs for patients with myelomeningocele. Gait Posture.

[14] Desloovere K, Molenaers G, Van Gestel L, Huenaerts C, Van Campenhout A, Callewaert B, Van de Walle P, Seyler J (2006). How can push-off be preserved during use of an ankle foot orthosis in children with hemiplegia? A prospective controlled study. Gait Posture.

[15] Bartonek A, Eriksson M, Gutierrez-Farewik EM (2007). Effects of carbon fibre spring orthoses on gait in ambulatory children with motor disorders and plantarflexor weakness. Dev Med Child Neurol.

[16] Collins SH, Kuo AD (2010). Recycling energy to restore impaired ankle function during human walking. PLoS One.

[17] Bregman DJ, van der Krogt MM, de Groot V, Harlaar J, Wisse M, Collins SH (2011). The effect of ankle foot orthosis stiffness on the energy cost of walking: a simulation study. Clin Biomech.

[18] Collins SH, Wiggin MB, Sawicki GS (2015). Reducing the energy cost of human walking using an unpowered exoskeleton. Nature.

[19] Bregman DJJ, Harlaar J, Meskers CGM, de Groot V. Spring-like ankle foot orthoses reduce the energy cost of walking in patients with reduced ankle push-off only when their stiffness is appropriate. Chapter 6 in thesis. The Optimal Ankle Foot Orthosis. ISBN 978–90–6464-486-3. 2011:105–24.

[20] Harper NG, Esposito ER, Wilken JM, Neptune RR (2014). The influence of ankle-foot orthosis stiffness on walking performance in individuals with lower-limb impairments. Clin Biomech (Bristol, Avon).

[21] *Aids to the examination of the peripheral nervous system*. London: Her Majesty’s Stationery Office; Medical Research Council, 1976.

[22] Beasley WC (1961). Quantitative muscle testing: principles and applications to research and clinical services. Arch Phys Med Rehabil.

[23] Cave EF, Roberts SM (1936). A method of measuring and recording joint function. J Bone Joint Surg.

[24] Jagadamma KC, Coutts FJ, Mercer TH, Herman J, Yirrel J, Forbes L, Van Der Linden ML (2009). Effects of tuning of ankle foot orthoses-footwear combination using wedges on stance phase knee hyperextension in children with cerebral palsy - preliminary results. Disabil Rehabil Assist Technol.

[25] Bregman DJ, Rozumalski A, Koops D, de Groot V, Schwartz M, Harlaar J (2009). A new method for evaluating ankle foot orthosis characteristics: BRUCE. Gait Posture.

[26] Kerkum YL, Brehm MA, Buizer AI, van den Noort JC, Becher JG, Harlaar J (2014). Defining the mechanical properties of a spring-hinged ankle foot orthosis to assess its potential use in children with spastic cerebral palsy. J Appl Biomech.

[27] Brehm MA (2007). The clinical assessment of energy expenditure in pathological gait [dissertation].

[28] Hausswirth C, Bigard AX, Le Chevalier JM (1997). The Cosmed K4 telemetry system as an accurate device for oxygen uptake measurements during exercise. Int J Sports Med.

[29] Garby L, Astrup A (1987). The relationship between the respiratory quotient and the energy equivalent of oxygen during simultaneous glucose and lipid oxidation and lipogenesis. Acta Physiol Scand.

[30] Kerkum YL, Buizer AI, van den Noort JC, Becher JG, Harlaar J, Brehm MA (2015). The effects of varying ankle foot orthosis stiffness on gait in children with spastic cerebral palsy who walk with excessive knee flexion. PLoS One.

[31] Russell Esposito E, Blanck RV, Harper NG, Hsu JR, Wilken JM (2014). How does ankle-foot orthosis stiffness affect gait in patients with lower limb salvage?. Clin Orthop Relat Res.

[32] Andrysek J, Klejman S, Kooy J (2013). Examination of knee joint moments on the function of knee-ankle-foot orthoses during walking. J Appl Biomech.

[33] Kerkum YL, Houdijk H, Brehm MA, Buizer AI, Kessels ML, Sterk A, van den Noort JC, Harlaar J (2015). The shank-to-vertical-angle as a parameter to evaluate tuning of ankle-foot orthoses. Gait Posture.

[34] Kobayashi T, Leung AK, Akazawa Y, Hutchins SW (2011). Design of a stiffness-adjustable ankle-foot orthosis and its effect on ankle joint kinematics in patients with stroke. Gait Posture.

[35] Kerkum YL, Brehm MA, van Hutten K, van den Noort JC, H J, Becher JG, Buizer AI (2015). Acclimatization of the gait pattern to wearing an ankle-foot orthosis in children with spastic cerebral palsy. Clin Biomech (Bristol, Avon).

[36] Gatts S (2008). Neural mechanisms underlying balance control in tai chi. Medicine and sport science.

