# Supplementary figures and images for: Stiffness modification of two ankle-foot orthosis types to optimize gait in individuals with non-spastic calf muscle weakness – a proof-of-concept study
Source: J Foot Ankle Res. 2019 Aug 7;12:41. doi: 10.1186/s13047-019-0348-8 (PMC6686412; doi:10.1186/s13047-019-0348-8)

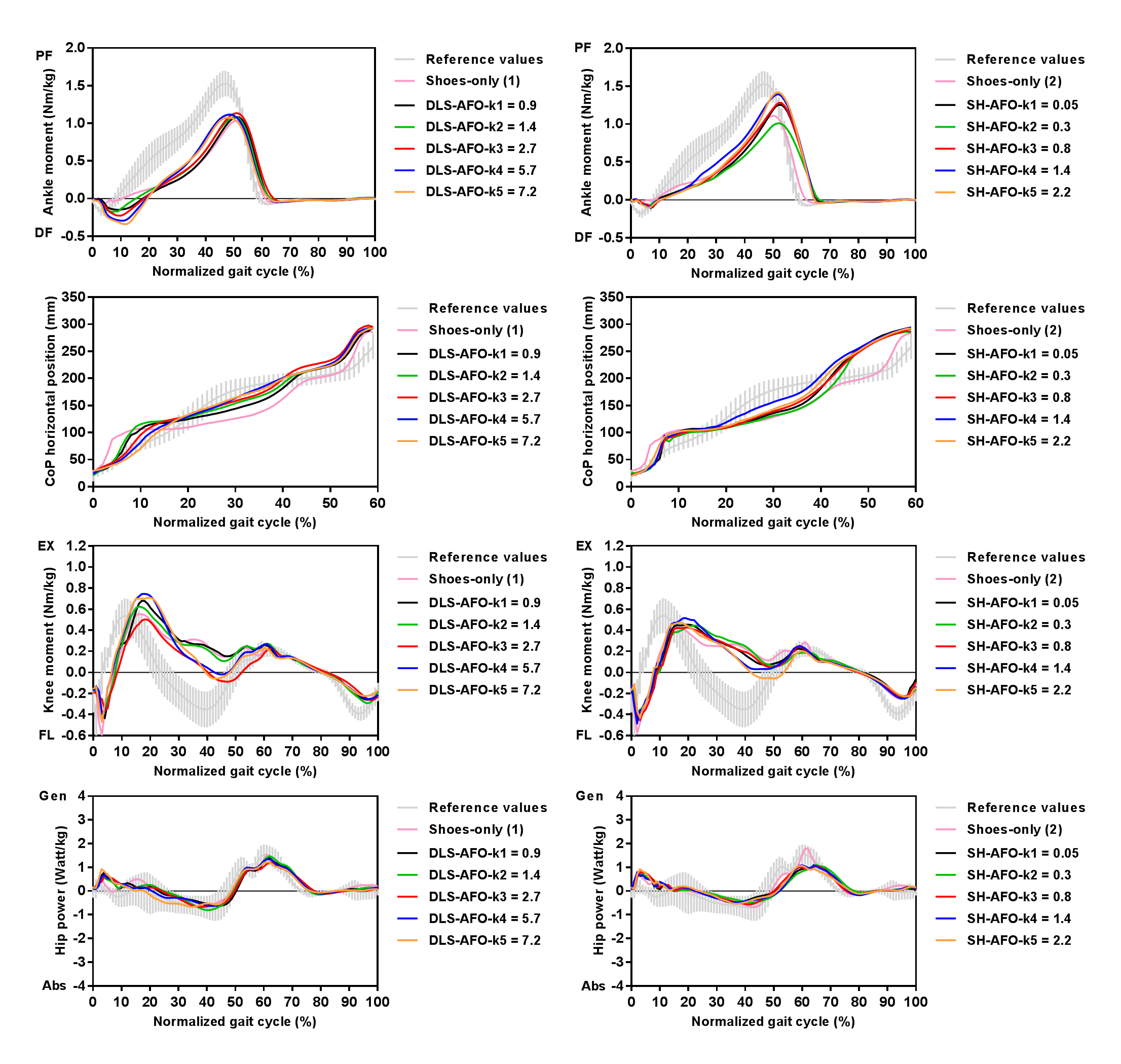

Supplement: Supplementary file 1 — Gait biomechanics of subject A (calf muscle strength MRC 4). Shoes-only (1) is performed at the DLS-AFO testing day, Shoes-only (2) is performed at the SH-AFO testing day. Abbreviations: DLS-AFO: dorsal-leaf-spring ankle-foot-orthosis, SH-AFO: spring-hinged ankle-foot-orthosis, k: stiffness in N•m•deg− 1, DF: dorsiflexion, PF: plantarflexion, EX: extension, FL: flexion, Gen: generation, Abs: absorption, CoP: center of pressure. (TIF 1005 kb) [file 13047_2019_348_MOESM1_ESM.tif]

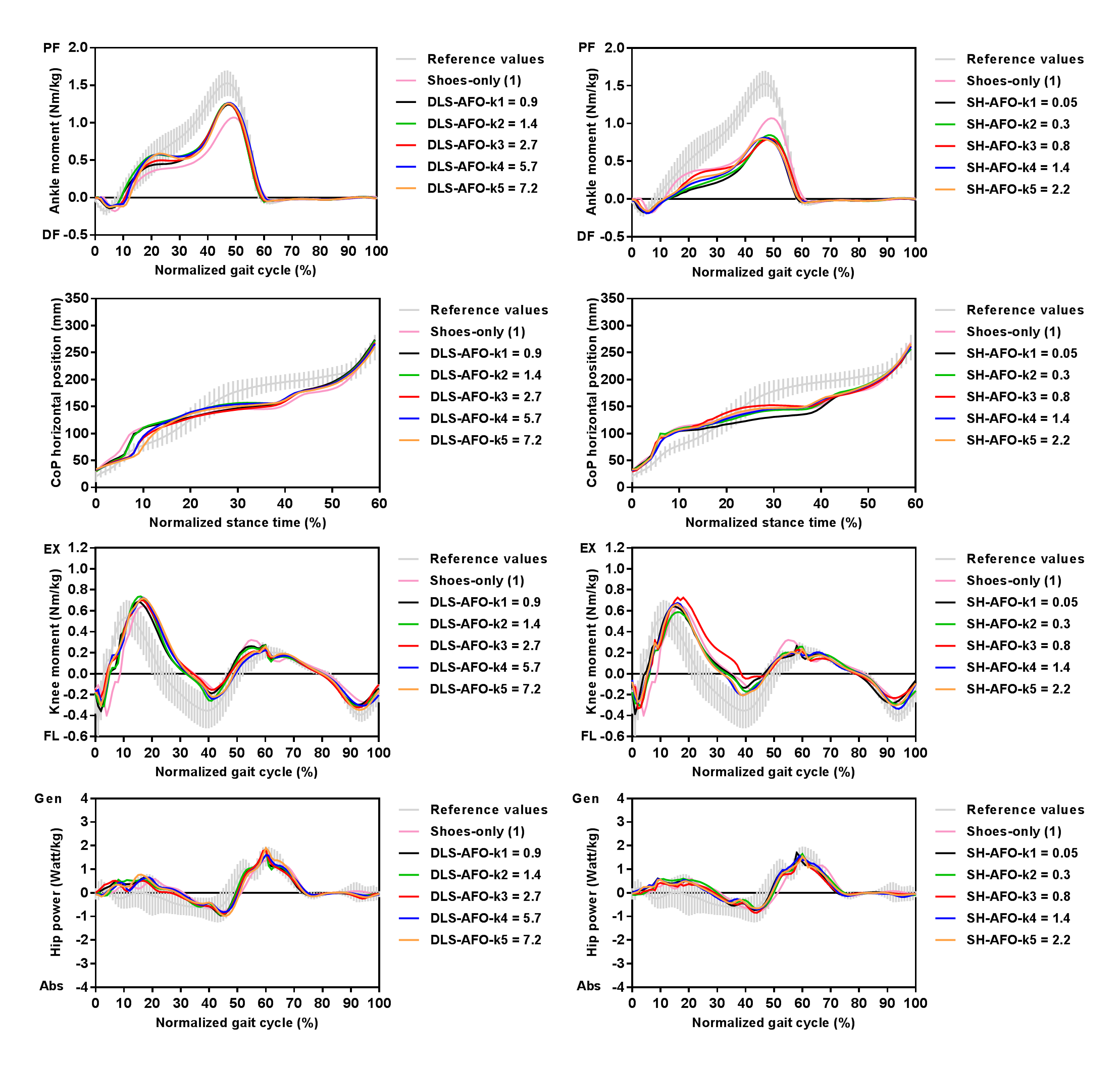

Supplement: Supplementary file 2 — Gait biomechanics of subject B (calf muscle strength MRC 4). Since all AFO conditions were tested at one day there is only one shoes-only (Shoes-only (1)) condition performed. Abbreviations: DLS-AFO: dorsal-leaf-spring ankle-foot-orthosis, SH-AFO: spring-hinged ankle-foot-orthosis, k: stiffness in N•m•deg− 1, DF: dorsiflexion, PF: plantarflexion, EX: extension, FL: flexion, Gen: generation, Abs: absorption, CoP: center of pressure. (TIF 995 kb) [file 13047_2019_348_MOESM2_ESM.tif]

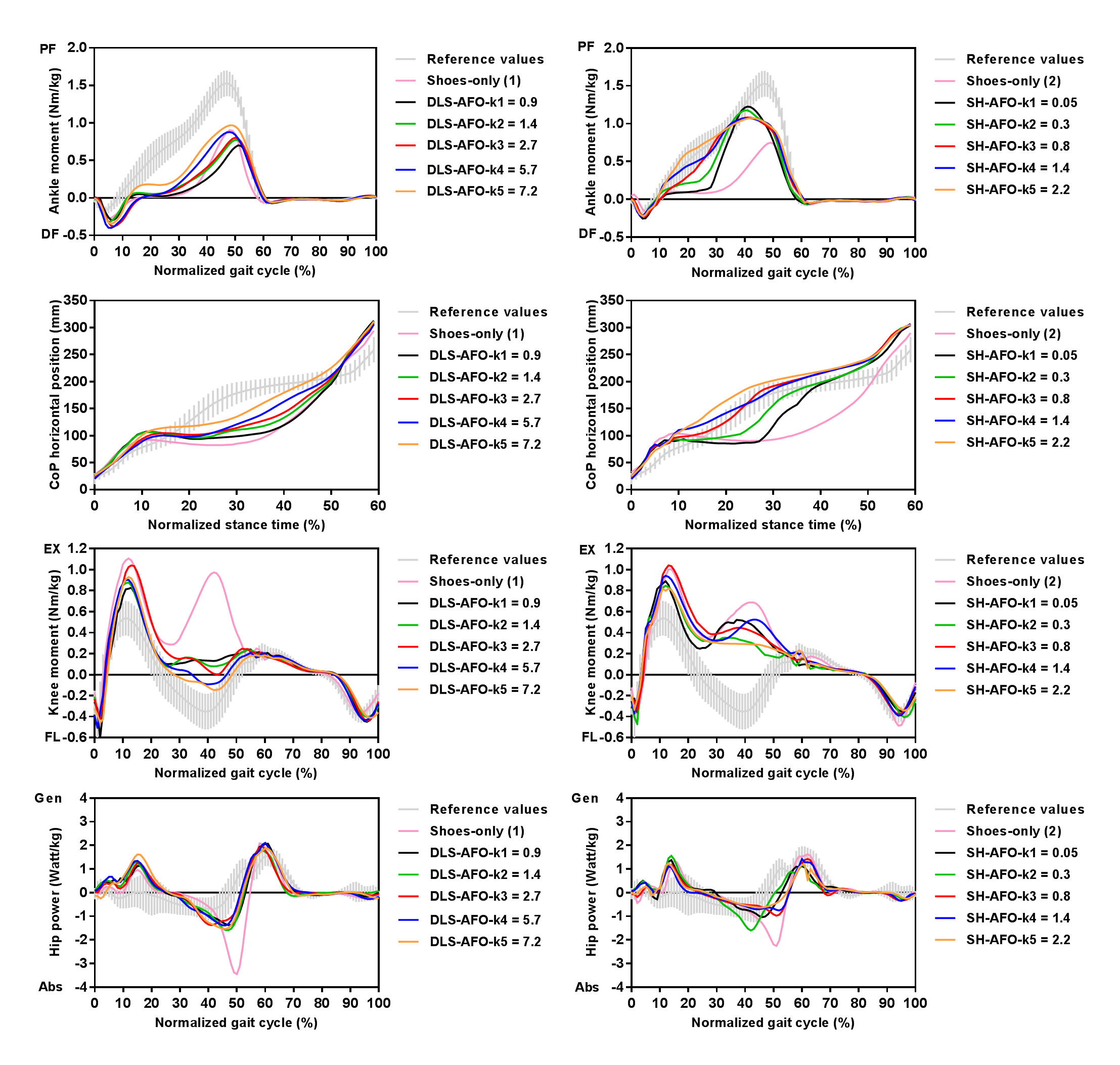

Supplement: Supplementary file 3 — Gait biomechanics of subject C (calf muscle strength MRC 0). Shoes-only (1) is performed at the DLS-AFO testing day, Shoes-only (2) is performed at the SH-AFO testing day. Abbreviations: DLS-AFO: dorsal-leaf-spring ankle-foot-orthosis, SH-AFO: spring-hinged ankle-foot-orthosis, k: stiffness in N•m•deg− 1, DF: dorsiflexion, PF: plantarflexion, EX: extension, FL: flexion, Gen: generation, Abs: absorption, CoP: center of pressure. (TIF 1057 kb) [file 13047_2019_348_MOESM3_ESM.tif]
